# Thioflavin-T does not report on electrochemical potential and memory of dormant or germinating bacterial spores

**DOI:** 10.1128/mbio.02220-23

**Published:** 2023-10-13

**Authors:** Yong-qing Li, Lin He, Makunda Aryal, James Wicander, George Korza, Peter Setlow

**Affiliations:** 1 School of Electrical Engineering and Intelligentization, Dongguan University of Technology, Dongguan, Guangdong, China; 2 Department of Physics, East Carolina University, Greenville, North Carolina, USA; 3 Department of Molecular Biology and Biophysics, UConn Health, Farmington, Connecticut, USA; University of Massachusetts Amherst, Amherst, Massachusetts, USA

**Keywords:** bacterial spore, spore germination, spore memory, electrochemical potential

## Abstract

Bacterial spores are metabolically dormant, resistant to microbicides, and vectors of food spoilage and diseases, while germinated spores are easy to kill. Consequently, understanding germination mechanisms may facilitate the development of “germinate-to-eradicate” strategies. Spores germinate in response to many compounds (called germinants). They can also retain the memory of a germinant exposure, such that a second exposure triggers more efficient germination, but how is not clear. A recent high-profile paper [Science (2022) 378:43] suggested that increasing spore electrochemical potential is how memory is “stored” based on measurements of *Bacillus subtilis* spores’ accumulation of the dye thioflavin-T after germinant exposure. Indeed, we found that wild-type spores of three *Bacillus* and one *Clostridioides* species all exhibited this early thioflavin-T accumulation during nutrient pulses. However, our data indicate that inferring spores’ electrochemical potential from thioflavin-T accumulation is problematic. We found that *B. subtilis* spores lacking their proteinaceous coat exhibited memory but did not accumulate thioflavin-T prior to germinant addition or during nutrient pulses. Furthermore, wild-type *Bacillus* spores germinating with dodecylamine, which also elicits memory, showed no thioflavin-T accumulation. Finally, we found that thioflavin-T accumulation by a germinating spore is outside the spore core at early stages but inside the spore core as germination proceeds. These findings suggest that thioflavin-T accumulation during the early stages of germination is due to its binding to one or more protein in the spore coat rather than to changes in spores’ electrochemical potential; thus, thioflavin-T is not a potentiometric dye for the study of spore memory of germinant pulses.

**IMPORTANCE:**

*Bacillus* and *Clostridium* spores cause food spoilage and disease because of spores’ dormancy and resistance to microbicides. However, when spores “come back to life” in germination, their resistance properties are lost. Thus, understanding the mechanisms of spore germination could facilitate the development of “germinate to eradicate” strategies. One germination feature is the memory of a pulsed germinant stimulus leading to greater germination following a second pulse. Recent observations of increases in spore binding of the potentiometric dye thioflavin-T early in their germination of spores led to the suggestion that increasing electrochemical potential is how spores “remember” germinant pulses. However, new work finds no increased thioflavin-T binding in the physiological germination of Coatless spores or of intact spores germinating with dodecylamine, even though spore memory is seen in both cases. Thus, using thioflavin-T uptake by germinating spores to assess the involvement of electrochemical potential in memory of germinant exposure, as suggested recently, is questionable.

## INTRODUCTION

Spores of bacterial species of Firmicutes are formed in sporulation which is commonly triggered by nutrient depletion. These spores are dormant and extremely resistant to multiple stress factors including heat, radiation, desiccation, antibiotics, and toxic chemicals (1). Consequently, spores can survive for years in the absence of nutrients and can be major vectors for food spoilage and serious diseases, including anthrax and severe diarrhea. However, when nutrients become available, spores can rapidly return to life in the process of germination and lose dormant spores’ extreme resistance properties (2). Consequently, germination could be spores’ “Achilles heel,” and indeed, there is ongoing work trying to develop “germinate to eradicate” strategies (3 – 5). Thus, it seems reasonable that this latter work might be facilitated by a detailed understanding of the mechanism(s) of spore germination.

Much is indeed known about the mechanisms of germination of spores of *Bacilli* and *Clostridia*, including that germination is most commonly triggered by the sensing of physiological germinants, which are small molecules, often sugars or amino acids, by spore inner membrane (IM) proteins termed germinant receptors (GRs); GRs can also be activated by moderate hydrostatic pressure (2). The association of germinants with GRs leads to the commitment of spores to germinate, and an early germination event is the release of a large fraction of the Na^+^, K^+^, and H^+^ from the spore’s central core (2, 6, 7) (8 – 10). These early changes trigger the release of the core’s depot of Ca^2+^ in a 1:1 chelate with dipicolinic acid (CaDPA) which comprises ~25% of core dry weight, with core water content as low as 25% of wet weight (1). CaDPA release is via IM channels composed of multiple SpoVA proteins whose opening is triggered by earlier germination events (1, 2, 11), and CaDPA release triggers hydrolysis of the large peptidoglycan (PG) cortex in *Bacillus* spores by two cortex-lytic enzymes (CLEs), CwlJ, and SleB. Cortex hydrolysis then allows both water uptake by the relatively dehydrated spore core and core swelling, increasing core wet weight as water to ~80%, the value in growing cells. This allows the resumption of spore metabolism including the production of ATP and macromolecular synthesis. Spores of *Bacilli* and some *Clostridia* can also trigger germination in response to germination triggers that are GR independent, including dodecylamine (DDA) that directly opens the SpoVA channel, as does high hydrostatic pressure (2).

In contrast to the germination of spores that have IM GRs, some *Clostridia* species, one being *Clostridioides difficile*, do not have IM GRs, but rather germinants are sensed by a protein in spores’ outer layers that proteolytically activates a CLE zymogen that hydrolyzes the cortex PG; this then leads to opening of the SpoVA channel and CaDPA release (2, 12).

A number of years ago, we found that spores of several *Bacilli* treated with a pulse of high pressure that activated GRs, followed by a second high-pressure pulse, exhibited a much higher germination response to the second pulse as if the first pulse had caused a change in the spores that did not germinate such that they were poised to respond to the second pulse (13). This finding was followed by a demonstration that spores of both *Bacilli* and *C. difficile* could generate memory of pulses of all small molecule germinants, including L-alanine or L-valine, dodecylamine, and CaDPA, as well as taurocholate plus glycine for *C. difficile* spores (14). Notably, the memory of the first pulse would decay if there was more time between the first and second pulses or if the spores were incubated at a higher temperature. The behavior noted above was termed spore memory, although how memory was stored and utilized was not determined.

Recently, however, the mechanism of spore memory of germinant exposure was re-examined in spores of *Bacillus subtilis* germinating with the GR-dependent germinant L-alanine (15). This study found that each nutrient pulse resulted in a change in the fluorescence intensity of the cationic dye thioflavin-T (ThT) that was presumably adsorbing to spores. Dyes like ThT can be used to monitor membrane potential based on their accumulation inside vegetative cells with negative membrane potential (16, 17). However, potentiometric dyes are generally unable to enter dormant spores and instead accumulate around the spore periphery. This peripheral fluorescence was assumed to report on spore membrane potential, and the stepwise increase in ThT fluorescence after each nutrient pulse was interpreted to reflect an increase in negative membrane potential due to the efflux of K^+^ ions (15). The authors concluded that partial K^+^ release and an increase in negative electrical potential are the mechanism by which spores retain the memory of a previous exposure to nutrients and when a threshold value of negative electrochemical potential is reached the spores germinate. However, the change in ThT fluorescence intensity (presumed to reflect a change in membrane potential) of an individual spore in a sufficient but long-germinant pulse or a constant germinant has not been measured, and it is unclear if an early increase in ThT fluorescence prior to CaDPA release must lead to spore germination. In addition, the memory model proposed based on ThT fluorescence changes (15) cannot easily account for previous observations that the memory of exposure to nutrients is lost over time or at a higher temperature (13, 14) and also cannot explain findings that GR-independent CaDPA or dodecylamine pulses, which do not work through IM GRs, can access memory generated by GR-dependent pulses and vice versa.

ThT has been used for decades as a probe for protein folding as this dye binds to beta sheets (18, 19). Since the spore coat is composed of >70 proteins, many of which are quite hydrophobic (20), we were concerned that peripheral staining by ThT was due to protein binding to elements in the hydrophobic spore coat rather than electrochemical potential. To investigate this possibility, in the current work, we analyzed ThT fluorescence in GR-dependent germination of wild-type (WT) spores of a number of species and *B. subtilis* spores lacking the protein coat and outer membrane (termed Coatless) and examined ThT binding during spores’ germination with the GR-independent germinant dodecylamine. As in previous work (15), we also found strong peripheral ThT fluorescence around WT spores that increased during the early stages of GR-dependent germination and prior to CaDPA release. However, (i) while WT and Coatless *B. subtilis* spores germinated in response to nutrients with similar kinetics and both exhibited spore memory, the Coatless spores were virtually unstained by ThT until after CaDPA release; and (ii) WT spores germinating with dodecylamine also exhibited no early ThT accumulation. These findings indicate that ThT cannot be used as a dye to measure germinating spores’ electrochemical potential and cannot generate data supporting a model based on electrochemical potential being the mechanistic basis of spore memory and exit from dormancy.

## RESULTS

### Coatless and WT *B. subtilis* spores retain the memory of nutrient pulses

**Fig 1:**
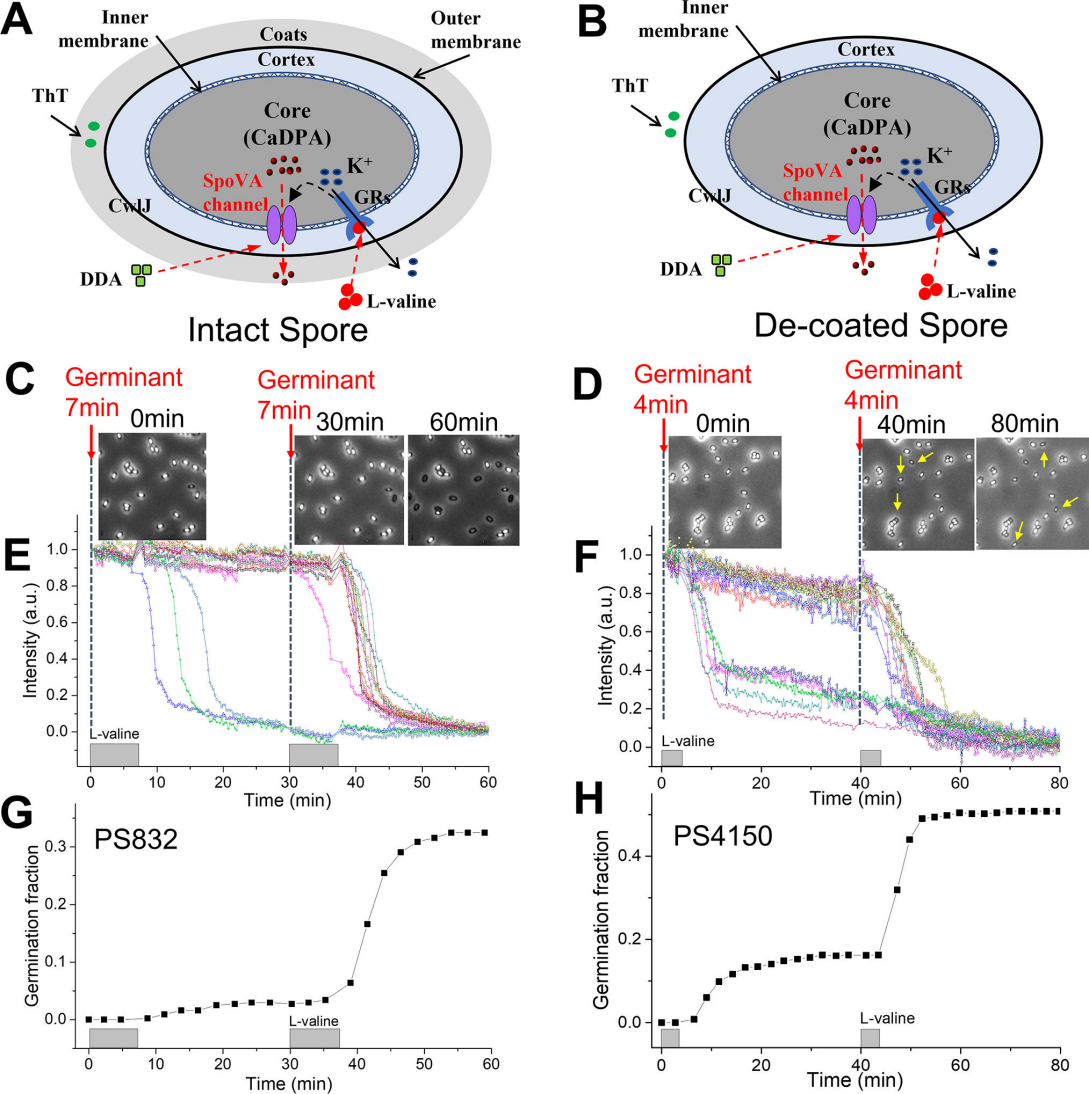
Spore memory is generated by stimulation of *B. subtilis* GRs in intact or Coatless spores. (**A and B**) Schematics of intact WT and Coatless spores and illustration of information flow in L-valine or dodecylamine germination. L-valine activates GRs in the IM, leading to the release of K^+^ via the GRs’ function as an ion channel (6), and somehow this opens the SpoVA channel for CaDPA release from the spore core. Dodecylamine directly opens the SpoVA channel allowing CaDPA release. Positively-charged ThT may bind to spore’s periphery, perhaps as a result of monovalent cation release and/or coat protein reorganization early in germination. (**C, E, and G**) Spores of *B. subtilis* PS832 (WT; Table 1) were given two 7-min 10 mM L-valine germinant pulses separated by 23 min at 37°C. (**D, F, and H**) Spores of PS4150 (Coatless; Table 1) were given two 4-min 10 mM L-valine pulses separated by 36 min at 37°C. Phase-contrast images were recorded at different times (**C and D**) at a rate of 15 s per frame, and changes in the normalized phase-contrast intensity of individual spores were traced during spore germination (**E and F**). The fraction of germination induced by each pulse was calculated as described in Materials and Methods (**G and H**). Gray bars above the horizontal axis indicate pulse durations. The yellow arrows in panel D indicate the germinated Coatless spores.

Fig. 1A and B show schematics of an intact and a Coatless *B. subtilis* spore and illustrate information flow during GR-dependent L-valine or non-GR-dependent dodecylamine germination. Fig. 1C through H show that spore memory can be generated in WT PS832 spores as well as in Coatless PS4150 spores (Table 1) as a result of GR stimulation by L-valine pulses which stimulate the same GR as L-alanine. There was ~ninefold more germination in the second L-valine pulse than in the first one in WT spores as found previously (14), and ~2.5-fold more germination in the second L-valine pulse than in the first one with coatless spores, a new finding. Previous work showed that *B. subtilis* spore memory can also be generated by pulses of the GR-independent germinant dodecylamine, which directly opens the SpoVA protein CaDPA channel, and germinant memory was also observed in *Bacillus cereus* and *C. difficile* spores (14).

**TABLE 1:** Bacterial strains

Species/strain	Phenotype	Source (reference)
*Bacillus subtilis* PS832^*a*^	WT	Laboratory 168 strain
*Bacillus subtilis* PS4150^*a*^	Coat-less	(21)
*Bacillus subtilis* PS4498^*a*^	GR-less	(22)
*Bacillus subtilis* FB113^*a*^	Cortex-lytic enzymeless	(23)
*Bacillus megaterium* QMB1551	WT	Hillel Levinson
*Bacillus cereus* T	WT	H.O. Halvorson
*Clostridioides difficile* ATCC43593	WT	American Type Culture Collection

^*a*^ All *Bacillus subtilis* strains are isogenic with PS832.

### During germination with a physiological germinant, ThT fluorescence increases prior to CaDPA release in *B. subtilis* WT and CLE-less spores (Table 1), but not in Coatless spores

**Fig 2:**
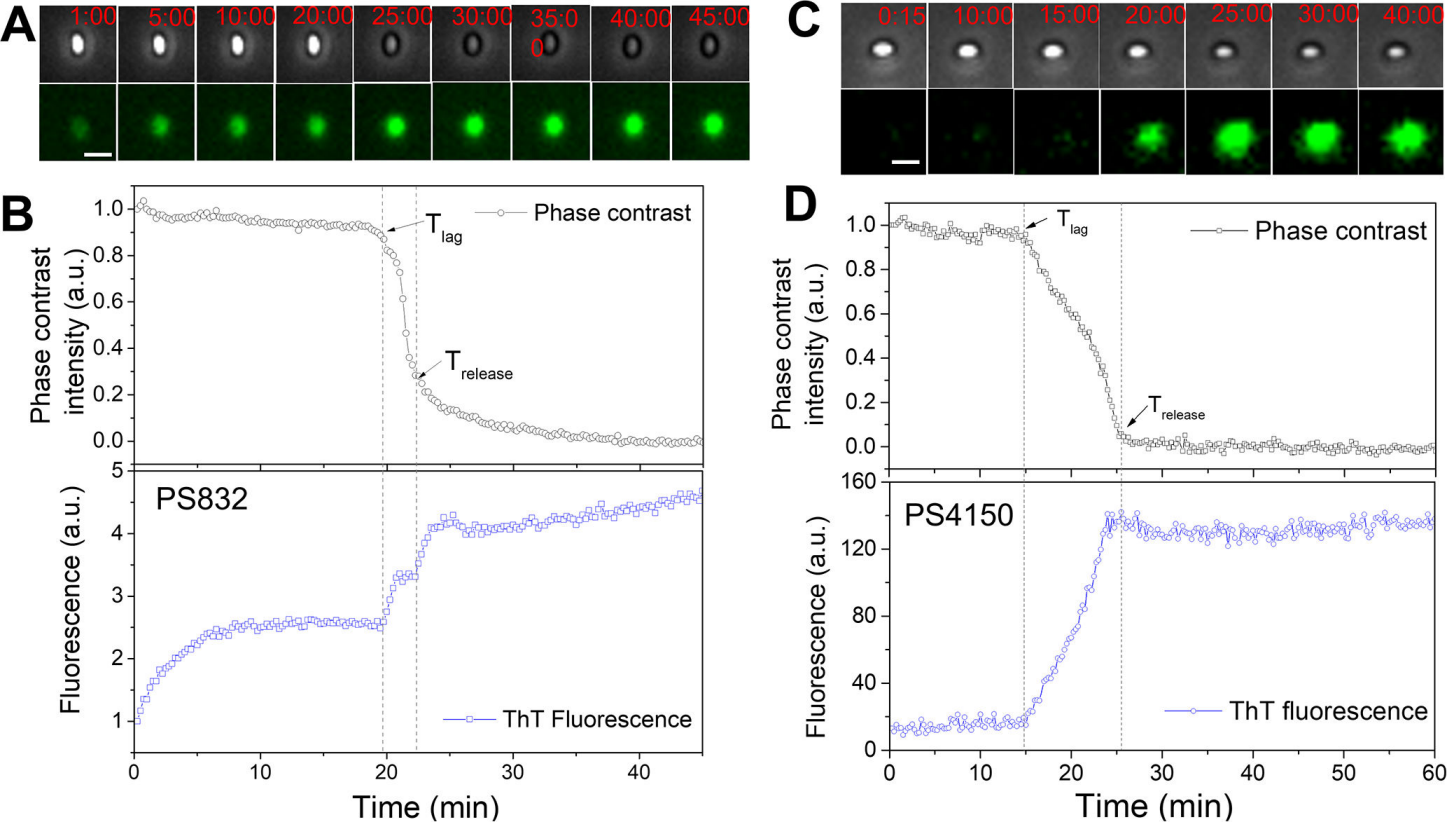
Germination of individual WT and Coatless (PS4150) *B. subtilis* spores with ThT/L-valine. (**A and C**) Phase contrast images (top panel) and fluorescence images (lower panel) obtained as described in Materials and Methods of a germinating (**A and B**) WT or (**C and D**) Coatless spore at different times after the addition of germinant. (**B and D**) Changes in normalized phase contrast intensity (upper panels) and ThT fluorescence intensity (lower panels) with incubation time after ThT/L-valine are added at time 0. Heat-activated spores were germinated with ThT/L-valine as described in Materials and Methods. The normalized fluorescence intensity was calculated as F_ThT_/F_ThT0_, where F_ThT0_ is ThT fluorescence at the resting state (the first image after the addition of germinant). The scale bar is 2 µm in panel A and 1.5 µm in panel C. The T_lag_ and T_release_ times noted with arrows are when rapid CaDPA release began and was finished, respectively.

Recently, spore memory of germinant exposure was confirmed in WT *B. subtilis* spores germinating with L-alanine, and it was found that each L-alanine pulse resulted in an increase in the fluorescence intensity of the cationic dye ThT, which was interpreted to reflect an increase in negative membrane potential due to efflux of K^+^ ions (15). However, it is unclear: (i) how ThT fluorescence of individual spores changes during germination with a constant germinant exposure, which presumably indicates the maximum accumulated change in electrochemical potential; and (ii) if an early increase in ThT fluorescence must lead to spore germination. Consequently, that ThT binding to germinating spores does indeed measure electrochemical potential needs validation. The results in Fig. 2A and B show that after the addition of L-valine plus 10 μM ThT at time 0, there is ~threefold increase in ThT fluorescence intensity of a WT *B. subtilis* spore, which remains fairly constant prior to T_lag_, the time for the start of the rapid release of all CaDPA from the spore core which finishes at T_release_, and is accompanied by a large decrease in the spore’s phase contrast image intensity due to CaDPA’s replacement by water. There is then a second increase in ThT fluorescence beginning at T_lag_ likely due to the concomitant release of CaDPA and uptake of ThT into the spore core (see below). Analysis of multiple individual WT spores germinating with L-valine plus ThT gave similar results (Fig. S1; Video S1). It should be noted that the time at which the ThT fluorescence increases to a constant value after the addition of ThT/germinant prior to rapid CaDPA release is nearly identical (~8 min) among individual germinating spores; however, the T_lag_ time is highly heterogeneous (Fig. S1), suggesting that the timing of CaDPA release by individual spores is independent of the initial increase in the ThT fluorescence. Fig. S2 shows L-valine germination of multiple individual CLE-less *B. subtilis* spores which can initiate germination and release CaDPA but not complete spore germination, and these spores also exhibited increases in ThT fluorescence prior to and after T_lag_. However, and importantly, the increase in ThT fluorescence prior to CaDPA release was not seen with Coatless *B. subtilis* spores germinating with L-valine, although the increase in ThT fluorescence starting at T_lag_ did take place (Fig. 2C and D; Fig. S3, Video S2). Since germinating Coatless spores do show memory (Fig. 1F through H ), these findings suggest that either the increase in ThT fluorescence prior to CaDPA release does not report on spore memory with a GR-dependent germinant, or spore membrane potential is not involved in spore memory.

### The early increase in ThT fluorescence occurs in germination with physiological germinants not only with *B. subtilis* spores, but also with *B. megaterium*, *B. cereus*, and *C. difficile* spores, but not with the GR-independent germinant dodecylamine

Increases in ThT fluorescence also occur prior to rapid CaDPA release in *B. megaterium* and *B. cereus* spores exposed to GR-dependent germinant (Fig. S7 and S8). As seen with the germination of spores of *Bacillus* species with GR-dependent germinants, multiple individual germinating *C. difficile* spores also exhibited a large increase in ThT fluorescence soon after physiological germinant addition and prior to rapid CaDPA release (Fig. S10). However, increases in ThT fluorescence prior to CaDPA release were not observed in dodecylamine germination of *B.subtilis* spores of various strains (Fig. S4 to S6), or with *B. megaterium* and *B. cereus* spores (Fig. S9). Notably, *Bacillus* spores germinating with dodecylamine also show memory of pulses of this GR-independent germinant (14). These findings certainly mean that ThT fluorescence cannot be used to monitor the membrane potential of spores germinating with dodecylamine.

### Ungerminated spores also exhibit and maintain an early increase in ThT fluorescence

**Fig 3:**
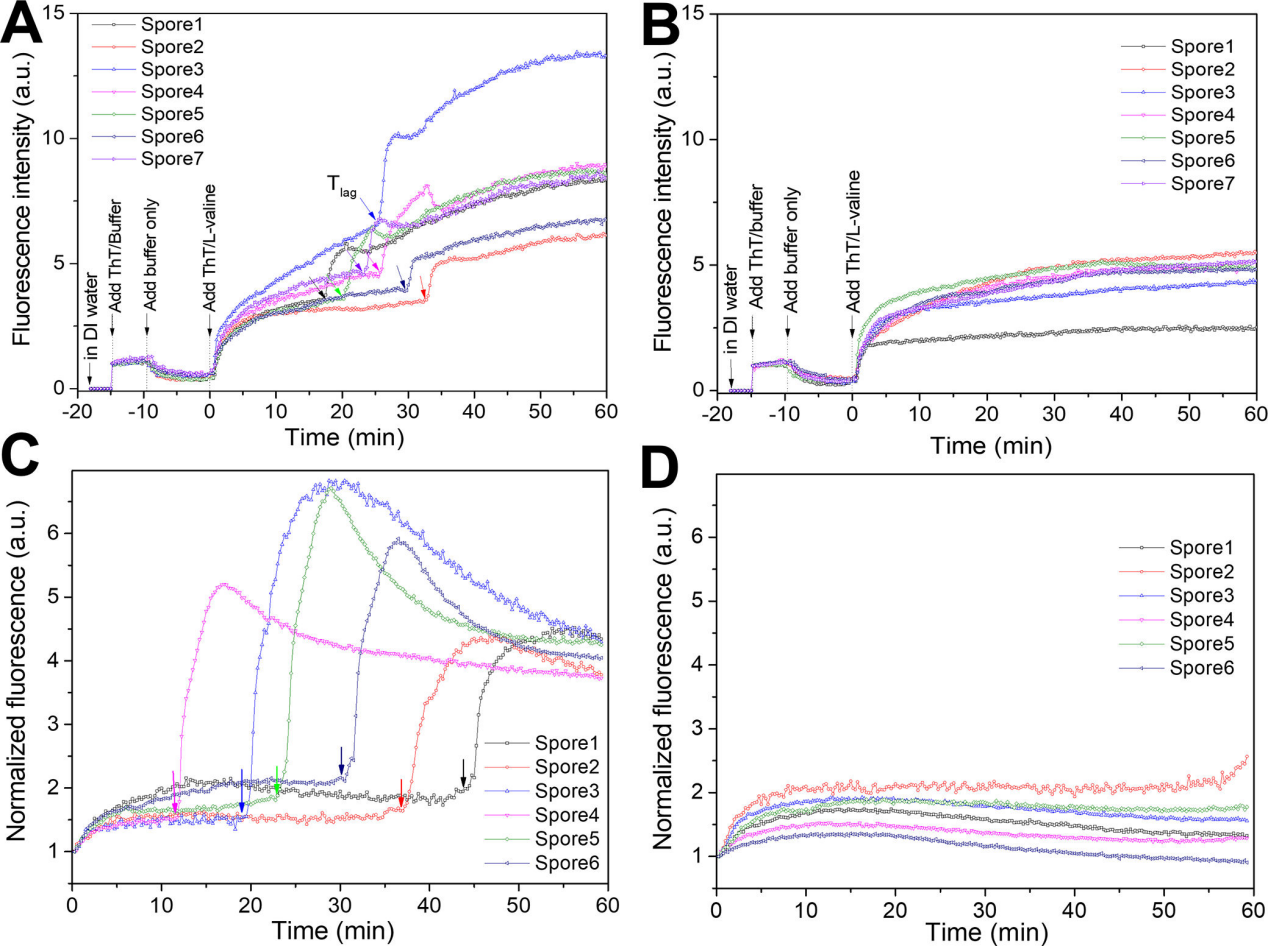
Changes in ThT fluorescence intensities of germinated and ungerminated individual WT *Bacillus* spores with ThT/germinant. (**A**) Fluorescence intensities of *B. subtilis* PS832 (WT) spores without heat activation that were incubated sequentially in distilled water, ThT/Hepes buffer, Hepes buffer only, and finally ThT/L-valine and that did germinate; arrows indicate T_lag_ times when rapid CaDPA release began. (**B**) Fluorescence intensities of *B. subtilis* spores incubated as in (A) but that did not germinate. (**C**) ThT fluorescence intensities of multiple *B. megaterium* spores that germinated in ThT/glucose; arrows indicate T_lag_ times. (**D**) ThT fluorescence intensities of multiple individual ungerminated spores of *B. megaterium* incubated in ThT/glucose. Heat-activated *B. megaterium* spores were germinated with 0.5 mM D-glucose and 10 µM ThT as described in Materials and Methods.

We also found that the increase in ThT fluorescence prior to Ca-DPA release in L-valine germination did not necessarily lead to spore germination. Fig. 3A and B show ThT fluorescence intensities of multiple individual PS832 (WT) *B. subtilis* spores incubated first in ThT plus buffer, then only buffer and then L-valine/ThT. After the addition of L-valine/ThT, the germinated spores exhibited the reported increase in ThT fluorescence (Fig. 3A). While there was a small increase in spore ThT fluorescence with ThT-buffer alone, with ThT/valine, the spore ThT fluorescence increased up to fivefold prior to CaDPA release at T_lag_. However, spores that failed to germinate exhibited similar early increases in ThT fluorescence but did not proceed to CaDPA release (Fig. 3B). Similarly, after the addition of ThT/glucose, while germinated spores of *B. megaterium* exhibited the early increase in ThT fluorescence (Fig. 3C), the spores that did not initiate germination exhibited similar increases in ThT fluorescence but without proceeding to CaDPA release (Fig. 3D). Again, the time at which the ThT fluorescence increases to a steady and maximum value prior to CaDPA release is short (~8 min) among individual germinating spores (Fig. 3C and D), although the T_lag_ time is highly heterogeneous among individual spores (Fig. 3C), suggesting that the time when rapid CaDPA begins, T_lag_, is independent of the timing of the ThT fluorescence increase. The initial increase in ThT fluorescence but not the one after T_lag_ was also observed and maintained in *B. cereus* spores incubated in ThT/germinant that did not proceed to germination (Fig. S8C). These findings further argue that the increase in ThT fluorescence prior to rapid CaDPA release may be unrelated to changes in spores’ electrochemical potential and are also likely to be unrelated to the commitment of spores to germinate and exit from dormancy.

### ThT accumulation after germinant addition is initially outside spore core but accumulates inside the core following CaDPA release

**Fig 4:**
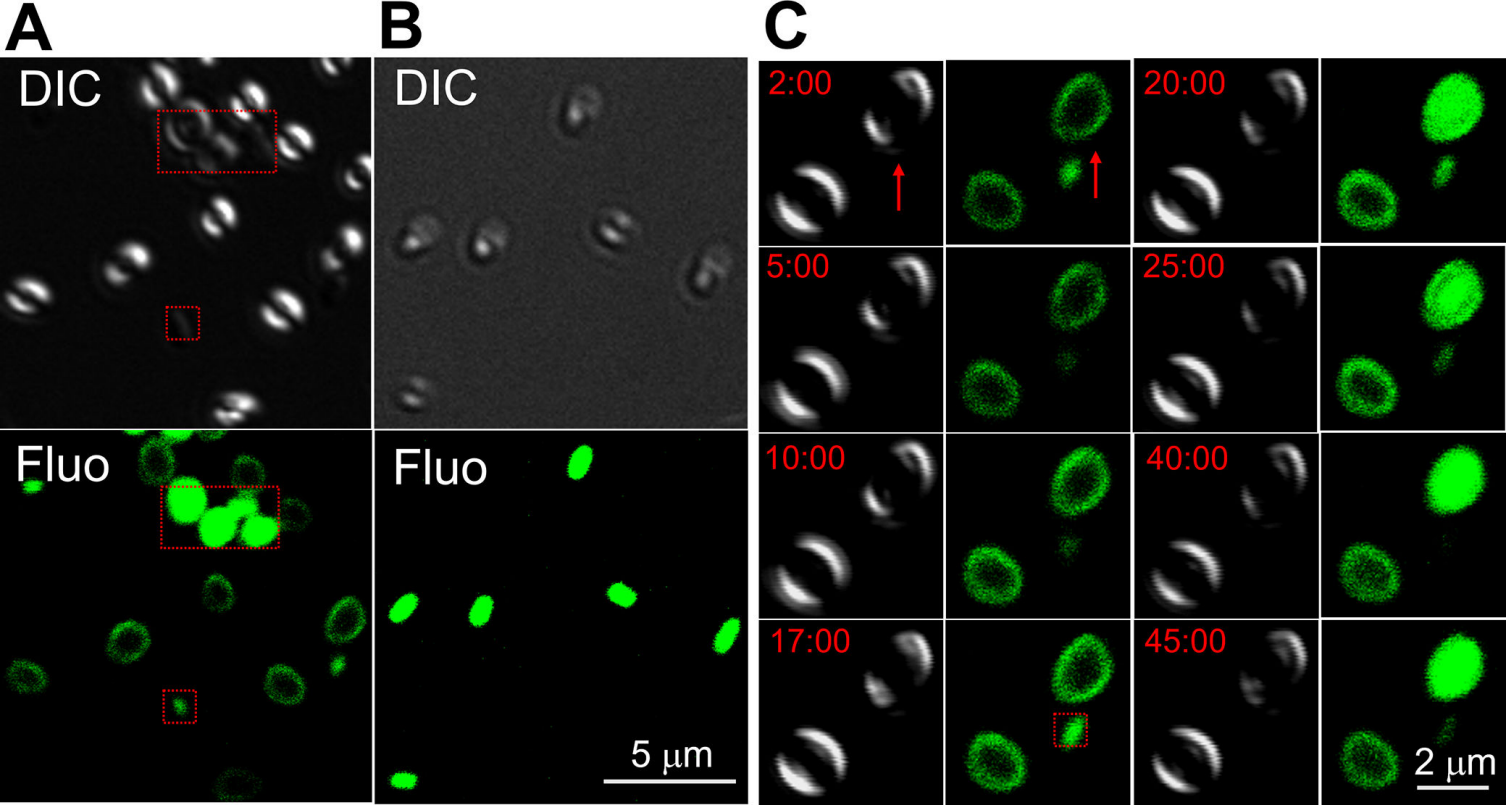
(A–C) LSCM of WT *B. megaterium* spores during germination with ThT/glucose. (**A**) DIC and ThT fluorescence images of dormant spores incubated in ThT/glucose for 1 min. (**B**) DIC and ThT fluorescence images of spores incubated in ThT/glucose for 60 min with all germinating. (**C**) Time-lapse DIC and ThT images of two spores incubated in ThT/glucose with one germinating (red arrows) and one that did not as described in Materials and Methods. The DIC images (which only show spores’ outer edges) appear bright for dormant spores and dark for germinated spores. Note that outer layer staining remains in the spore that germinated in panel C, as can be seen best in the 20- and 25-min time points. Some cell debris and germinated spores existed in panel A (boxed in red), and cell debris existed in panel C (boxed in red) due to imperfect sample preparation.

Previous studies have found that ThT and other potentiometric dyes cannot enter dormant spores and only accumulate around the spore periphery (24, 25), but whether ThT enters spores upon germination has not been investigated. Laser-scanning confocal fluorescence microscopy (LSCM) images showed that ThT fluorescence was initially only in the outer region of dormant spores of *B. megaterium* after the addition of ThT/glucose (Fig. 4A). However, the dye was primarily located in the core in spores following initiation of rapid CaDPA release, although the outermost staining remained (Fig. 4B and C). Fig. 4C shows time-lapse differential interference contrast (DIC) and ThT fluorescence images of both a germinating *B. megaterium* spore (red arrow) and a non-germinating spore. After the addition of ThT/glucose, the DIC images were initially bright, indicating both spores are dormant, and the ThT fluorescence was in spores’ outer layer. After 5 min, there was a noticeable increase in both spores’ ThT fluorescence intensity, but the fluorescence was still only in spores’ outer region. At ~20 min, the DIC image of one spore (red arrow) became dark, indicating the release of CaDPA from the core, and the ThT fluorescence was brighter and largely in the spore core, although the outer layer staining remained (Fig. 4C, see 20 and 25 min images). This finding is consistent with the data in Fig. S7 but with much better spatial resolution due to the confocal imaging in Fig. 4. Note, also in Fig. 4C, that between 20 and 45 min, the spore that had not germinated had essentially the same outer layer staining with ThT as did the spore that fully germinated starting at 20 min. These results confirm that: (i) the increase in ThT fluorescence in dormant spores is initially due to ThT binding to the spores’ outer layer, which includes the coat plus crust; (ii) the large increase in ThT fluorescence beginning soon after commencement of rapid CaDPA release is due to ThT accumulation in the germinating spore core; and (iii) spores with high outer layer ThT staining may still not germinate.

### Dodecylamine prevents ThT binding to an intact WT *B. subtilis* spore’s outer layers

**Fig 5:**
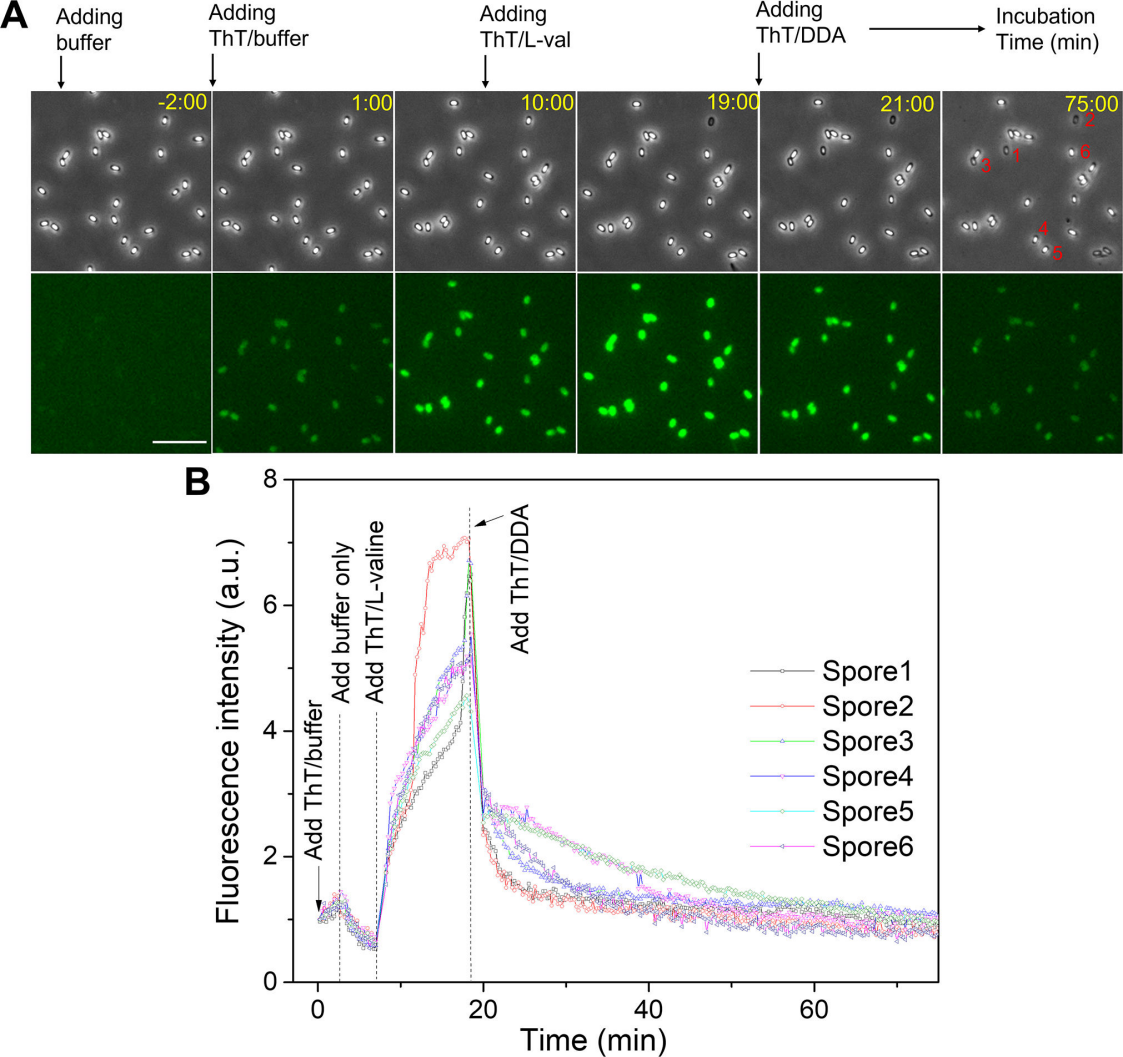
Changes in ThT fluorescence of multiple individual WT *B. subtilis* spores when ThT/L-valine was replaced by ThT/dodecylamine. (**A**) Time-lapse phase contrast and fluorescence images at different times before and after spores’ exposure to ThT/L-valine and then ThT/dodecylamine, as described in Materials and Methods; the scale bar is 10 µm. (**B**) Changes in ThT fluorescence intensities of multiple individual spores vs time. PS832 (WT) spores (no heat activation) were first incubated in ThT/buffer [25 mM Hepes buffer, (pH 7.4) and 10μM ThT], the ThT/buffer removed and spores incubated in the buffer for ~5 min. The buffer was then replaced by ThT/L-valine (10 mM L-valine and 10μM ThT in 25 mM Hepes) for ~10 min and then replaced by ThT/dodecylamine (1.0 mM dodecylamine and 10μM ThT in 25 mM Hepes) for ~60 min, as described in Materials and Methods. ThT fluorescence intensities in arbitrary units (a.u.) were normalized to the first image intensity. Three germinated spores [note the rapid rise in ThT fluorescence of these three spores in (**B**)] are labeled 1–3, and three ungerminated spores (no sharp rises in ThT fluorescence) are labeled 4–6 in (**A and B**).

As noted above, *B. subtilis* spores acquire memory after pulses of a GR-dependent, and when exposed to ThT/L-valine, *B. subtilis* spores exhibit an increase in ThT fluorescence (Fig. 2B). If ThT fluorescence is indeed reporting spores’ electrical potential, then the ThT signal is predicted to increase after each germinant pulse until reaching a threshold value to activate germination as proposed in a recent model (15). In the context of this model, a pulse of the germinant dodecylamine, which also elicits spore memory, should increase spores’ electrical potential (and by extension ThT fluorescence) until causing CaDPA release. However, when exposed to ThT/dodecylamine, germinated spores exhibited no significant ThT fluorescence (Fig. S4). Similarly, we also found that the addition of dodecylamine reduced the ThT fluorescence caused by a previous ThT/L-valine pulse (Fig. 5). These findings argue further that the ThT fluorescence does not report on spores’ electrical potential.

We further observed that after the addition of ThT/buffer, the outer surface of dormant WT PS832 *B. subtilis* spores was immediately weakly stained by ThT (Fig. 5A; Fig. S11). It was observed that some ThT accumulation of dormant spores in ThT/buffer occurred in the first 10–15 min before reaching a leveling off (Fig. S11) and replacement of ThT/buffer with buffer alone only led to a drop in ThT fluorescence (Fig. 5B; Fig. S11). The addition of ThT/L-valine then caused the reported large increase in ThT fluorescence (Fig. 5B), while the germinated spores had an additional increase in ThT fluorescence due to CaDPA release and core ThT uptake (e.g., spores 1–3 in Fig. 5B). The removal of ThT/L-valine and incubation in ThT/dodecylamine gradually decreased the ThT fluorescence intensities for both ungerminated spores and germinated spores to the initial level seen in ThT/buffer (Fig. 5A and B). This finding suggests that the addition of dodecylamine displaced ThT from a dormant spore’s periphery, consistent with the absence of ThT fluorescence prior to Ca-DPA release in dodecylamine germination (Fig. S4 and S9). Further decreases in ThT fluorescence by dodecylamine in spores that have lost most CaDPA may be because dodecylamine most likely permeabilizes germinated spores leading to spore death (26).

## DISCUSSION

Membrane potential is the difference in electrical potential across a cell membrane and plays an important role in bacterial function (16, 17). A small but significant early increase in ThT fluorescence of *B. subtilis* spores in response to an L-alanine germinant pulse was recently reported and was ascribed to an increase in spores’ electrochemical potential due to intracellular K^+^ release, and spores that required multiple early germinant pulses to trigger germination exhibited a multistep progressive increase in ThT fluorescence (15). We have largely duplicated this result and showed similar early ThT fluorescence increases in germinating spores of several other *Bacillus* and one *Clostridioides* species. Notably, there is monovalent cation release early in the germination of spores of the other *Bacillus* species (2), but whether this is also true of *C. difficile* spore germination is not known. The electrochemical potential model (15) to explain spore memory of germinant exposure(s) relied on measurements of the electrochemical potential of dormant and germinating spores based on the accumulation of the cationic fluorescent dye ThT. This dye has been used previously by the Süel group (27, 28) as a probe to monitor electrochemical potential in *B. subtilis* cells, based on its accumulation inside cells with negative membrane potential. However, here, we show that early increased ThT fluorescence (i.e., prior to CaDPA release) in germinating spores may not report on the membrane potential of dormant and germinating spores, as ThT is unable to enter dormant spores but only accumulates around germinating spore’s outer region prior to CaDPA release. More importantly, we also showed that Coatless *B. subtilis* spores that are germination-competent and display memory of a previous germinant exposure accumulated minimal if any ThT in germination prior to CaDPA release nor did intact spores of several species germinating with dodecylamine, a germinant that again generates memory of its exposures (14). In addition, ungerminated WT *Bacillus* spores exhibited and maintained the levels of ThT fluorescence similar to those in spores that germinated (Fig. 3B and D; 4C), indicating that changes in spores leading to the increase in ThT accumulation prior to CaDPA release alone are not sufficient to drive spores to germinate. All these results lead to two possible conclusions: one is that if early ThT accumulation by germinating spores is indeed due to changes in spores’ electrical potential, then electrical potential alone has no essential role in all spore memory; the second is that ThT accumulation early in germination is not a response to changes in spore electrical potential, but rather due to ThT binding to one or several hydrophobic spore coat proteins perhaps exposed by coat reorganization early in the germination process. Whichever of these conclusions is correct, the measurements of ThT binding to spores do not provide data to support a model for spore memory based on changes in spores’ electrochemical potential.

Finally, there are other possible explanations for spore memory, as follows. One is that recent work suggests that GRs oligomerize into nutrient germinant-gated ion channels. Based on these findings, the memory of a previous germinant exposure could be explained by partial occupancy of the GR oligomer after germinant exposure. However, this model requires that debinding of germinants from GRs be rather slow, and this has not yet been verified experimentally. A second is that the multi-protein SpoVA channel for CaDPA release in germination may comprise a cation-gated channel that is activated by the monovalent cation release prior to CaDPA release in germination (14), although again, this has not been verified experimentally nor has dodecylamine germination via activating the CaDPA-channel been shown to cause monovalent cation release. Clearly, there is still work to be done to unravel the mystery of spore memory of germinant exposures.

## MATERIALS AND METHODS

### Bacterial strains used, spore preparation, and germinant used

All bacterial strains of spore-formers used in this work are listed in Table 1.

PS4498, GR-less, a PS832 derivative that lacks all five of this species’ operons that encode GRs was constructed from PS832 by iterative transformation with chromosomal DNA from strains carrying a deletion replacement with a GR operon replaced by an antibiotic resistance marker and subsequent removal of the antibiotic genes by use of the Cre recombinase (29). All *Bacillus* spores were prepared and purified as described previously (30 – 33), and the final step was centrifugation through a high-density solution of Histodenz in which dormant spores pellet while cells, germinated spores and debris float. The *C. difficile* spores were prepared and purified as described previously (34).

The germinants used in this work were: (i) 10 mM L-valine in 25 mM Hepes (pH 7.4) at 37°C or 1.0 mM dodecylamine in 25 mM Hepes (pH 7.4) at 45°C for *B. subtilis* spores; (ii) 0.5–10 mM D-glucose in 25 mM KPO_4_ buffer (pH 7.4) at 30°C or 1.0 mM dodecylamine in 25 mM Hepes (pH 7.4) at 45°C for *B. megaterium* spores; (iii) 10 mM L-alanine in 25 mM Hepes (pH 7.4) at 37°C or 0.8 mM dodecylamine in 25 mM Hepes (pH 7.4) at 25°C for *B. cereus* spores; (iv) 0.25% taurocholate and 10 mM glycine in 10 mM Tris-HCl (pH 8.0) at 37°C for *C. difficile* spores. Prior to germination with nutrient germinants, *Bacillus* spores were heat activated by incubation in water and then cooled on ice for at least 15 min; specific heat activation conditions were: 70°C for 30 min with WT- or GR-less *B. subtilis* spores and 30 min for Coatless PS4150 spores, and 65°C for 30 min for *B. cereus* and *B. megaterium* spores. No heat activation is needed for either dodecylamine germination or *C. difficile* spore germination with taurocholate plus glycine (35, 36).

### Spore memory of germinant pulses

Analysis of spore memory of germinant exposures was essentially as described in previous work (14, 37). Briefly, heat-activated *B. subtilis* spores were spread on the surface of a glass coverslip that was coated with 0.01% poly-l-lysine (Sigma Aldrich) and sealed on a sample holder kept at a constant temperature. The spores were germinated with L-valine as described above by adding the germinant at T_0_ for 4–7 min before the germinant was removed, and spores were rinsed three times with germination buffer using a vacuum pump suction (14) and then incubated at 37°C in germination buffer for 23–36 min. Then, the spores were given a second exposure to germinants at 37°C in 25 mM germination buffer (pH 7.4) followed by germinant removal, rinsing by vacuum pump suction and further incubation at 37°C in germination buffer (pH 7.4). Phase contrast images of hundreds of individual spores were recorded with a charge-coupled device (CCD) camera at a rate of 15 s per frame. The number of spores that were germinated by the first pulse was defined as N_germ1_, that by the second pulse was defined as N_germ2_, and the total number of spores examined was defined as N_0_. The percentage of spores that germinated in the first pulse was (N_germ1_/N_0_) ×100% and that in the second pulse was N_germ2_/(N_0_ - N_germ1_) ×100%.

### Live-cell fluorescence microscopy and phase contrast microscopy of multiple individual germinating spores

A drop of spore suspension (2 µL, ~10^8^ spores/mL in water) was placed on the surface of a poly-l-lysine-coated coverslip and placed in a vacuum desiccator for ~5 min to dry the spore film. The microscope sample holder with the coverslip-adhered spores was mounted on a temperature-controlled thermal platform of an inverted microscope (Nikon Ti) which contains an external phase-contrast system and an immersion objective (Plan Apo 60×, NA1.4). The solution (~300 µL) of a mixture of germinant and ThT (10 µM) was added at time 0 of incubation, and fluorescence and phase contrast images were acquired simultaneously at a rate of 15 s per frame for 1–2 hours (24, 38). The illumination light for phase contrast microscopy was a computer-controlled white light-emitting diode (LED) source, and the excitation light for fluorescence microscopy was a computer-controlled blue LED (470 nm). A digital CCD camera (1,392 × 1,040 pixels and 12 bits) was used to capture the phase-contrast images, and an EMCCD camera (Andor, iXon Ultra 897, 512 × 512 pixels, 16 bits) was used to acquire the ThT fluorescence images with a filter (Semrock, FF01-520/35). The phase contrast LED was programmed to turn off when acquiring the fluorescence images (with an acquisition time of 500 ms), and the blue LED was turned off when acquiring phase contrast images (with an acquisition time of 300 ms) to avoid interference of phase contrast and fluorescence imaging. A home-made auto-focus system was developed to actively lock in the focus of the objective by using a diode laser at 650 nm to detect the distance change between the objective and the surface of the sample coverslip, which was fed back to a piezo attached on the objective to lock its position (38).

Both the phase contrast and fluorescence images were analyzed with a computation program in Matlab to locate each spore’s position and to calculate the averaged pixel intensity of an area of 16 × 16 pixels that covered the whole individual spore on the phase contrast/fluorescence images; this was calculated after subtraction of the background intensity of a nearby spore-free area. The phase contrast image intensity of each individual spore was plotted as a function of the incubation time, and the initial intensity at T_0_ (the first phase contrast image recorded after the addition of the germinant) was normalized to 1 and the intensity at the end of measurements was normalized to zero. This analysis allowed the determination of germination timing parameters of multiple individual spores, including the: lag time T_lag_ before rapid CaDPA release began, although there is some CaDPA leakage prior to T_lag_ (1, 2); T_release_, the time when CaDPA release is complete; and T_lys_, the time for the completion of cortex lysis and core swelling. The precise correspondence of T_lag_ and T_release_ in phase contrast imaging to CaDPA release was determined by Raman spectroscopy (24, 38). The fluorescence image intensity of each individual spore was also plotted as a function of the incubation time such that changes in ThT fluorescence can be compared to the germination timing or event determined by phase contrast microscopy (24)

### LSCM and DIC microscopy of multiple individual *B*. *megaterium* spores during germination

A drop of heat-activated *B. megaterium* spores (2 µL, ~10^8^ spores/mL in water) was placed on the surface of a poly-l-lysine-coated coverslip, vacuum dried, and mounted on an inverted LSCM microscope (Olympus FV1000) which contains a DIC system, an autofocus zero drift correct system, an immersion objective (60×, NA1.42) and an air-temperature control system. Germinant solution (~500 µL) of 1 mM glucose and 10 µM ThT in 25 mM KPO_4_ (pH 7.4) was added and incubated at 30°C. An excitation laser at 458 nm was used to scan across multiple germinating spores to generate confocal images (512 × 512 pixels, covering 20 × 20 µm) at the emission wavelength of 505–550 nm. To achieve the best axial resolution, the size of the confocal pinhole was set as 50 µm. The confocal fluorescence images and DIC images were acquired at a rate of 2 min per frame for ~60 min.

### Time-lapse fluorescence microscopy of multiple individual *B. subtilis* spores when ThT/L-valine was replaced with ThT/dodecylamine

A drop of PS832 (WT) *B. subtilis* spores (without heat activation) was placed on the surface of a poly-l-lysine-coated coverslip, vacuum dried, and mounted on a temperature-controlled thermal platform of an inverted microscope. While acquiring the ThT fluorescence images of multiple individual spores with an EMCCD camera at a rate of 15 s per frame, the spores were first incubated at 37°C in ThT/buffer (25 mM Hepes buffer10 µM ThT) for ~2 min, ThT/buffer removed, then spores incubated in buffer only for ~5 min, then in ThT/L-valine (10 mM L-valine, 10 µM ThT, and 25 mM Hepes) for ~10 min, and then in ThT/dodecylamine (1 mM dodecylamine, 10 µM ThT, and 25 mM Hepes) for ~60 min. Changes in ThT fluorescence intensities of individual spores were calculated as a function of incubation time.

## Data Availability

All data are available in the main text or the supplementary materials.

## SUPPLEMENTAL MATERIAL

Supplemental material mbio.02220-23-s0001.docx Supplemental text, Fig. S1–S11, and captions for Videos S1 and S2.

Video S1 mbio.02220-23-s0002.avi Germination of multiple individual WT *B. subtilis* (PS832) spores with L-valine/ThT. Heat-activated spores were germinated with L-valine/ThT added at time 0 and examined by phase contrast and ThT fluorescence microscopy as described in Materials and Methods. Movie S1 shows time-lapse phase contrast images (left in the movie) and ThT fluorescence images (right in the movie) that were recorded at a rate of 15 s per frame for ~60 min.

Video S2 mbio.02220-23-s0003.avi Germination of multiple individual coatless *B. subtilis* (PS4150) spores with L-valine. Heat-activated spores were germinated with L-valine/ThT added at time 0 and examined by phase contrast and ThT fluorescence microscopy as described in Materials and Methods. Shown are time-lapse phase contrast images (left in the movie) and ThT fluorescence images (right in the movie) that were recorded at a rate of 15 s per frame for ~60 min.

ASM does not own the copyrights to Supplemental Material that may be linked to, or accessed through, an article. The authors have granted ASM a non-exclusive, world-wide license to publish the Supplemental Material files. Please contact the corresponding author directly for reuse.
