# Supplementary material for: Thioflavin-T does not report on electrochemical potential and memory of dormant or germinating bacterial spores
Source: mBio. 2023 Oct 13;14(5):e02220-23. doi: 10.1128/mbio.02220-23 (PMC10653816; doi:10.1128/mbio.02220-23)
Supplement: Supplemental material — Supplemental text, Fig. S1–S11, and captions for Videos S1 and S2. [file mbio.02220-23-s0001.docx]

Supplementary Materials for

**Thioflavin T does not report on electrochemical potential and memory of dormant or germinating bacterial spores**

Yong-qing Li^1,2*^, Lin He^1^, Makunda Aryal^2^, James Wicander^3^, George Korza^3^, and Peter Setlow^3*^

^1^School of Electrical Engineering and Intelligentization, Dongguan University of Technology, Dongguan, Guangdong, China.

^2^Department of Physics, East Carolina University, Greenville, NC 27858-4353, USA.

^3^Department of Molecular Biology and Biophysics, UConn Health, Farmington, CT 06030-3305, USA.

*Corresponding author. Yong-Qing Li, liy@ecu.edu; Peter Setlow, setlow@uchc.edu

**This PDF file includes:**

Supplementary Text

Figs. S1 to S11

Movies S1 to S2

**Other Supplementary Materials for this manuscript include the following:**

Supplementary Text

**Results**

**Increases in ThT fluorescence prior to Ca-DPA release are not observed in dodecylamine germination of *B. subtilis* spores of various strains**

In contrast to the increases in spores’ ThT fluorescence prior to T_lag_ in L-valine germination of spores with an intact coat, addition of the non-GR-dependent germinant dodecylamine gave no noticeable increase in ThT fluorescence prior to T_lag_ with wild-type (WT) (PS832), GR-less (PS4498) or Cortex Lytic Enzyme-less (FB113) spores, and only a minimal increase even after Tlag (Fig. S4, S5, S6). Given that WT *B. subtilis* spores do exhibit memory of dodecylamine pulses, the model for spore memory based on use of the ThT fluorescent probe to measure spore electrochemical potential (1) seems at odds with results with dodecylamine as the germinant.

**Increases in ThT fluorescence also occur prior to rapid Ca-DPA release in *B. megaterium* and *B. cereus* spores exposed to GR-dependent germinants but not dodecylamine**

The prior work on ThT accumulation by germinating spores used *B. subtilis* spores, and an obvious question is whether results are the same with spores of other *Bacillus* species. Consequently, we examined ThT accumulation by germinating *B. cereus* and *B. megaterum* spores (Fig. S7, S8). As seen with WT *B. subtilis* spores, *B, megaterium* and *B. cereus* spores germinating with ThT plus an appropriate GR-dependent germinant also exhibited an ~ 2-fold increase in ThT fluorescence prior to T_lag_ (Fig.S7A-C); Fig. S8) and then a large increase in ThT fluorescence after T_lag_ due to uptake of ThT into the spore core. Unlike GR germination with *B. megaterium* and *B. subtilis* spores where the increase in ThT fluorescence after T_lag_ remained high, the level of of ThT taken up after T_lag_ in *B. cereus* spores then rapdly fell to approximately the level seen prior to T_lag_ (Fig. S8B). However, as with *B. subtilis* spores germinating with dodecylamine, *B. cereus* spores germinating with dodecylamine also exhibited no notable increase in ThT fluorescence until T_lag_, but the subseqent rapid increase in ThT fluorescence did not decrease, as was the case with alanine germination (Fig. S9).

**ThT fluorescence increases** **prior to rapid Ca-DPA release in germinating *C. difficile* spores**

As seen with germination of spores of *Bacillus* species with GR-dependent germinants, multiple individual germinating *C. difficile* spores also exhibited a large increase in ThT fluorescence soon after germinant addition (Fig. S10A,B). Following release of CaDPA from the spore core (black curves in Fig. S10B), there were a further large changes in ThT fluorescence intensity including some rapid decreases (Fig. S10A,B). Note that *C. difficile* spores do not have IM GRs, but rather their GRs are in spores’ outer layer and their activation triggers a cascade that leads to cortex degradation and only then is CaDPA released via similar SpoVA channels as in *Bacillus* spores (2).

**References:**

1. Kikuchi K, Galera-Laporta L, Weatherwax C, Lam JY, Moon EC, Theodorakis EA, Garcia-Galvo J, Süel GM. 2022. Electrochemical potential enables dormant spores to integrate environmental signals. Science 378:43-49.
2. Setlow P, Wang S, Li YQ. 2017. Germination of spores of the orders *Bacillales* and *Clostridiales*. Annu. Rev. Microbiol. 71:459-477.

Fig. S1.


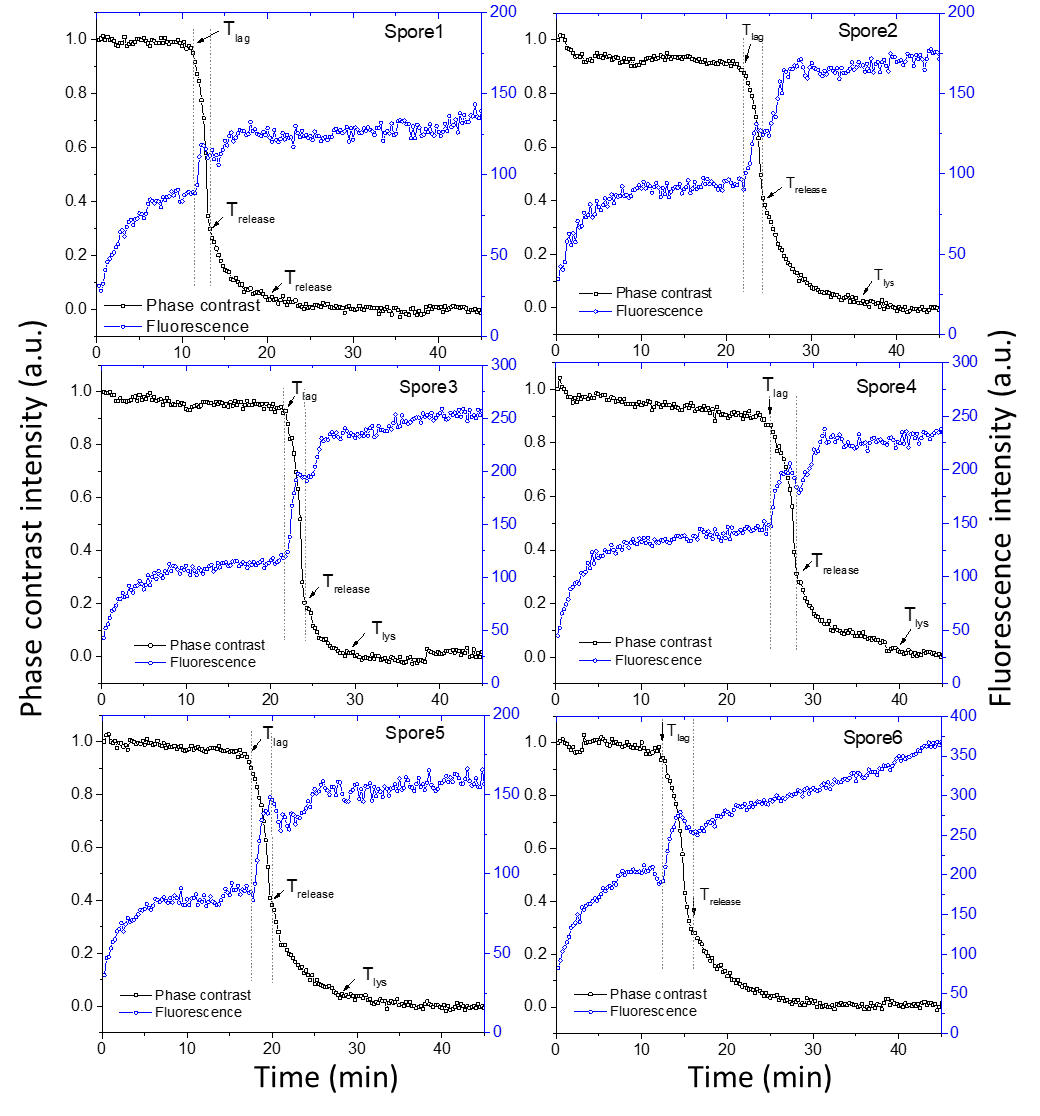
 **Fig. S1**. **Germination of multiple** **individual PS832 WT *B. subtilis* spores with L-valine/ThT.** Heat-activated spores were germinated with L-valine/ThT added at time 0 and examined by phase contrast and ThT fluorescence microscopy as described in Methods. Phase contrast intensities in arbitrary units (a.u.) were normalized to the first image intensity. The dashed grey lines indicate T_lag_ and T_release_ times.

Fig. S2.


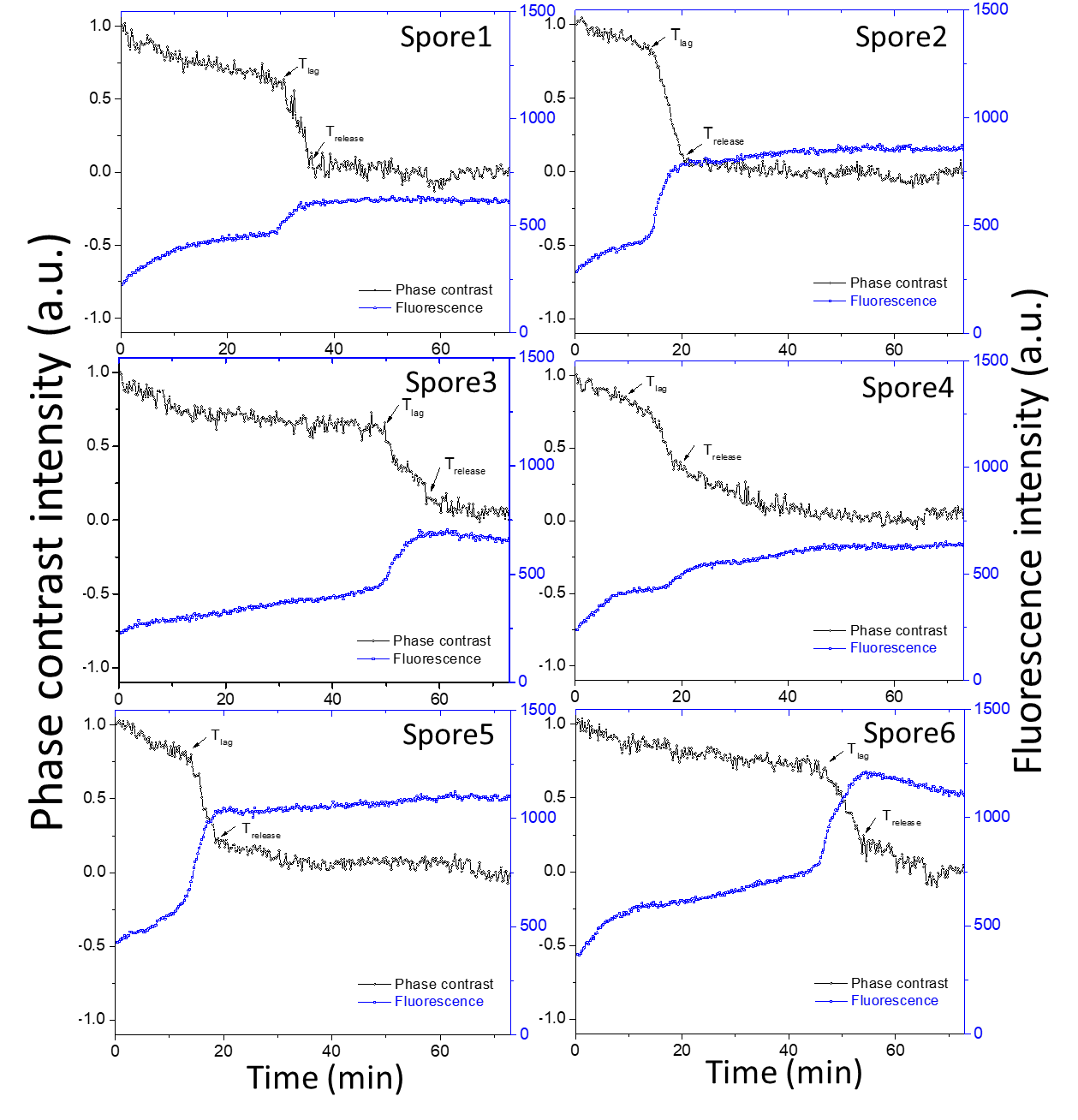
 **Fig. S2. Germination of multiple individual CortexLyticEnzyme-less (FB113) *B. subtilis* spores with L-valine/ThT.** Spores were germinated with L-valine/ThT added at time 0 and observed with phase contrast and fluorescence microscopy as described in Methods. Phase contrast intensities in arbitrary units (a.u.) were normalized to the first image intensity. The arrows indicate T_lag_ and T_release_ times.

Fig. S3.


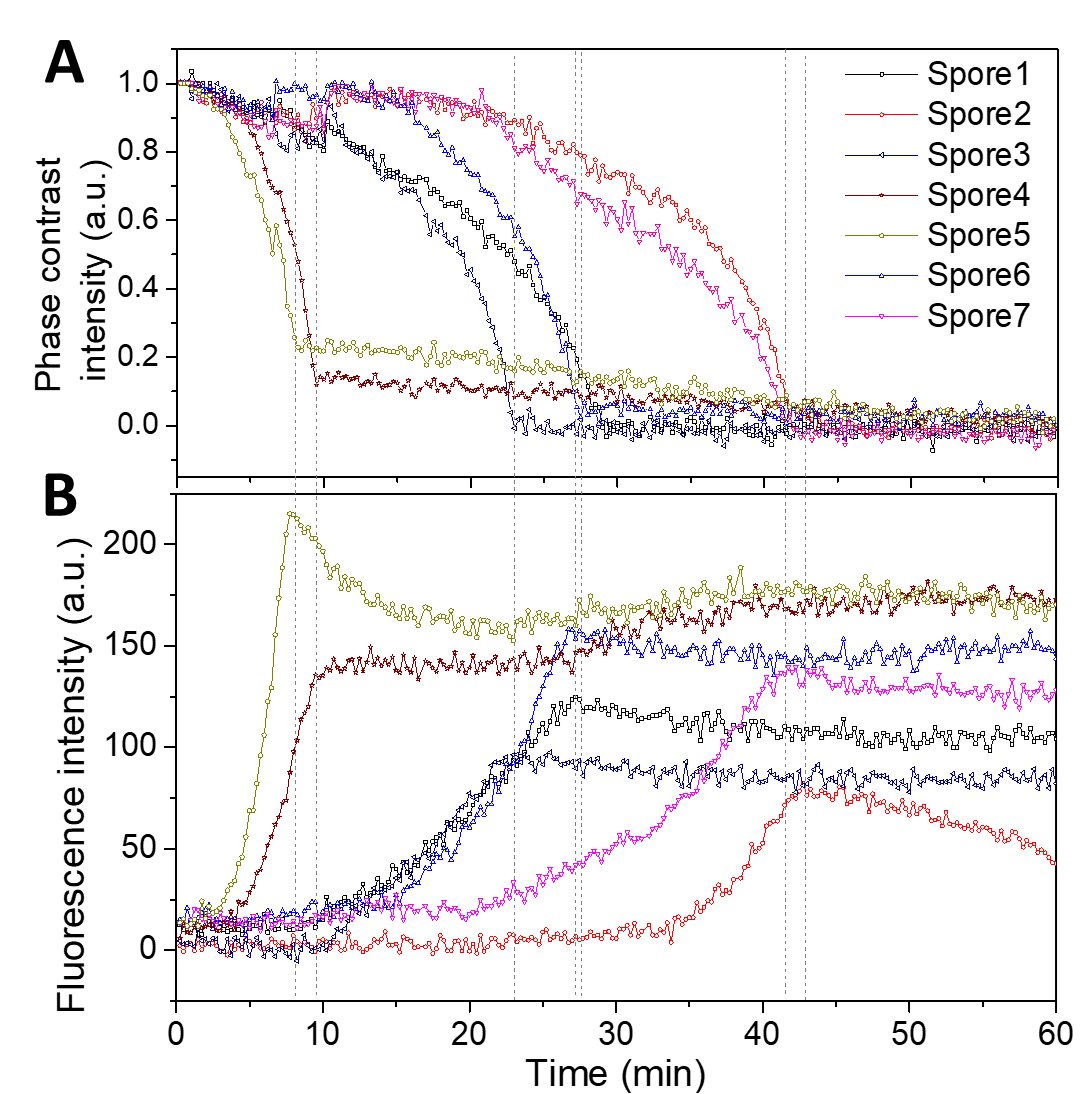


**Fig. S3**. **Germination of multiple individual PS4150 Coatless *B. subtilis* spores with L-valine.** Spores were germinated with L-valine and ThT added at time 0 and examined by phase contrast and fluorescence microscopy as described in Methods. **(A)** Normalized intensity of phase contrast images vs incubation time; **(B)** Intensities of ThT fluorescence images vs incubation time. The phase contrast intensities in arbitrary units (a.u.) were normalized to the first image intensity. The dashed grey lines indicate T_release_ times for individual spores.

Fig. S4.

**
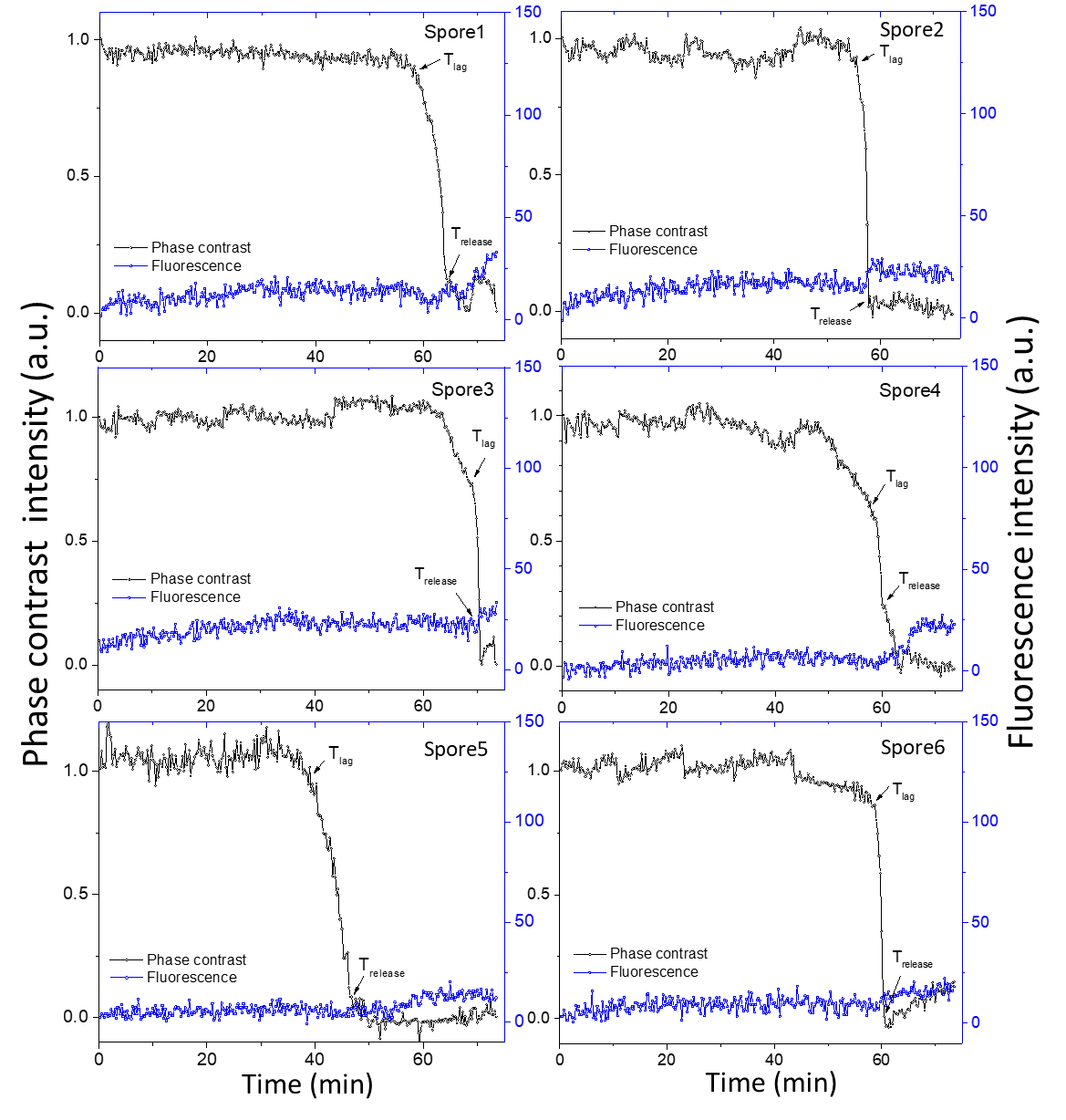
Fig. S4.** **Germination of multiple individual PS832 WT *B. subtilis* spores with dodecylamine.** Spores were germinated with dodecylamine and ThT added at time 0, and observed with phase contrast and fluorescence microscopy as described in Methods. The phase contrast intensities in arbitrary units (a.u.) were normalized to the first image intensity. The arrows indicate T_lag_ and T_release_ times, respectively.

Fig. S5.


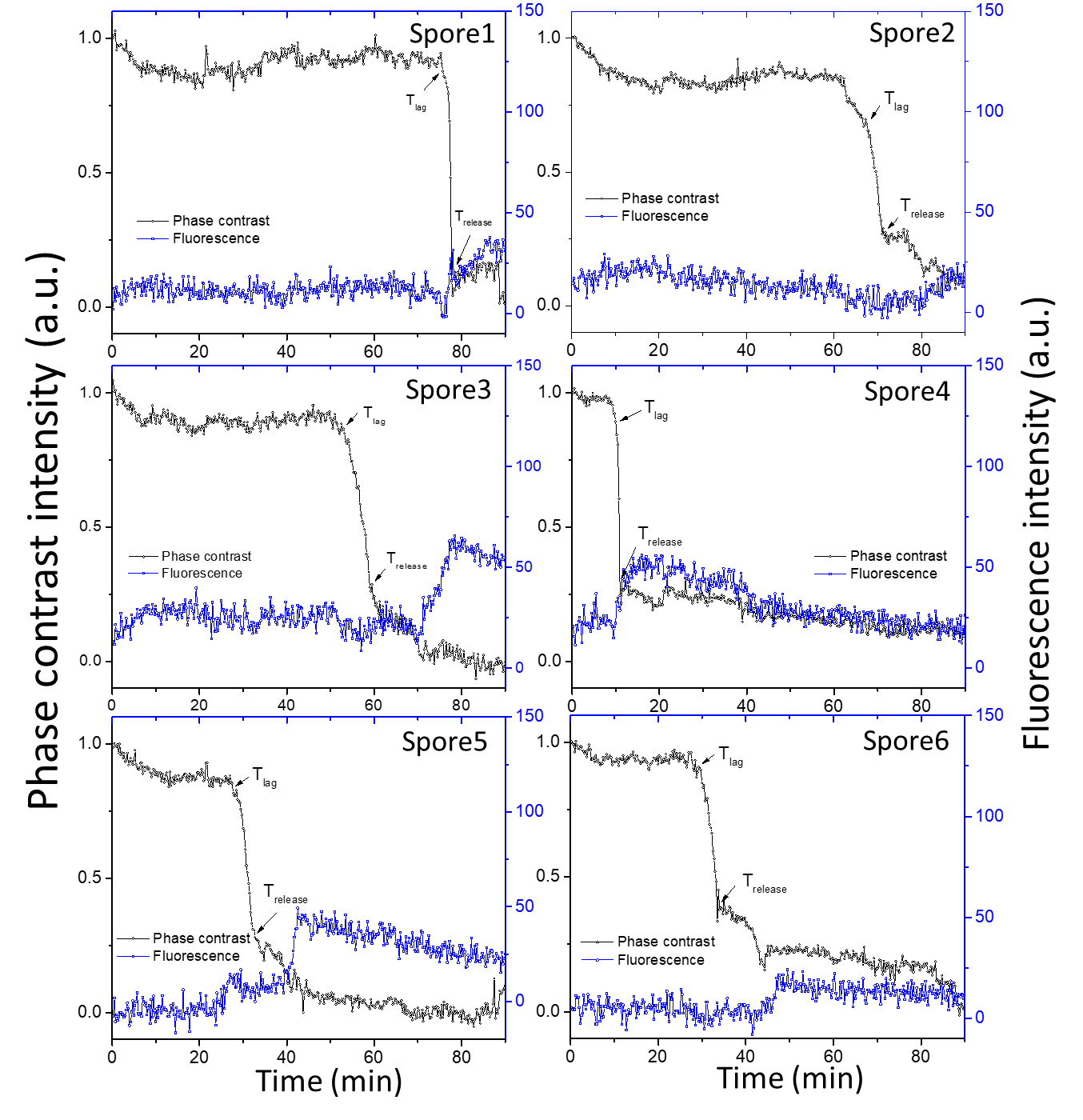
**Fig. S5.** **Germination of multiple individual GR-less (PS4498) *B. subtilis* spores with dodecylamine.** Spores were germinated with ThT/dodecylamine added at time 0, and observed by phase contrast and fluorescence microscopy as described in Methods. Phase contrast and fluorescence intensities in arbitrary units (a.u.) were normalized to the first image intensity. The arrows indicate T_lag_ and T_release_ times.

Fig. S6.


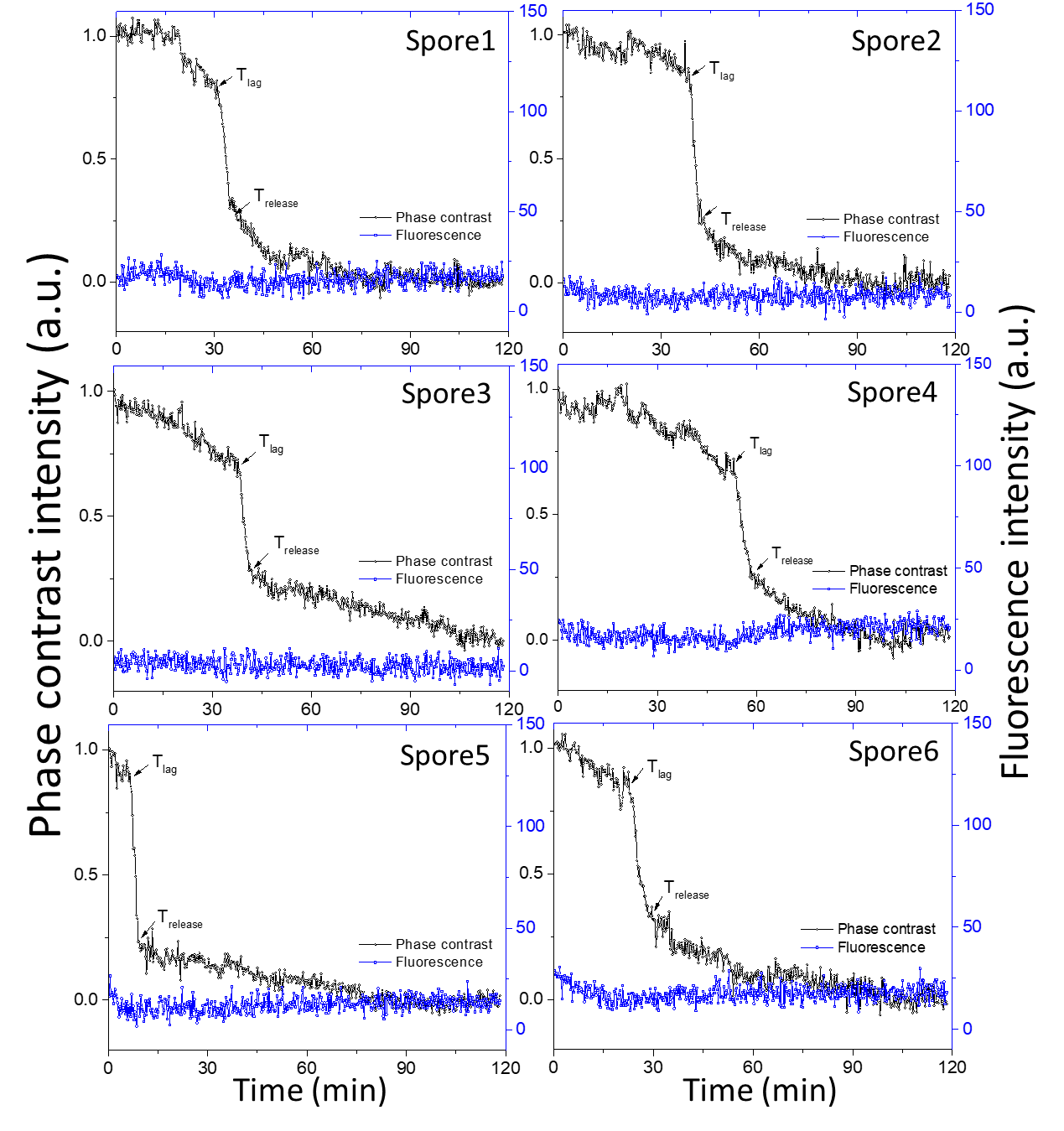
**Fig. S6.** **Germination of multiple individual CortexLyticEnzyme-less (FB113) *B. subtilis* spores with dodecylamine.** Spores were germinated with ThT/dodecylamine added at time 0 and observed by phase contrast and fluorescence microscopy as described in Methods. The phase contrast and fluorescence intensities in arbitrary units (a.u.) were normalized to the first image intensity. The arrows indicate T_lag_ and T_release_ times.

Fig. S7.

**
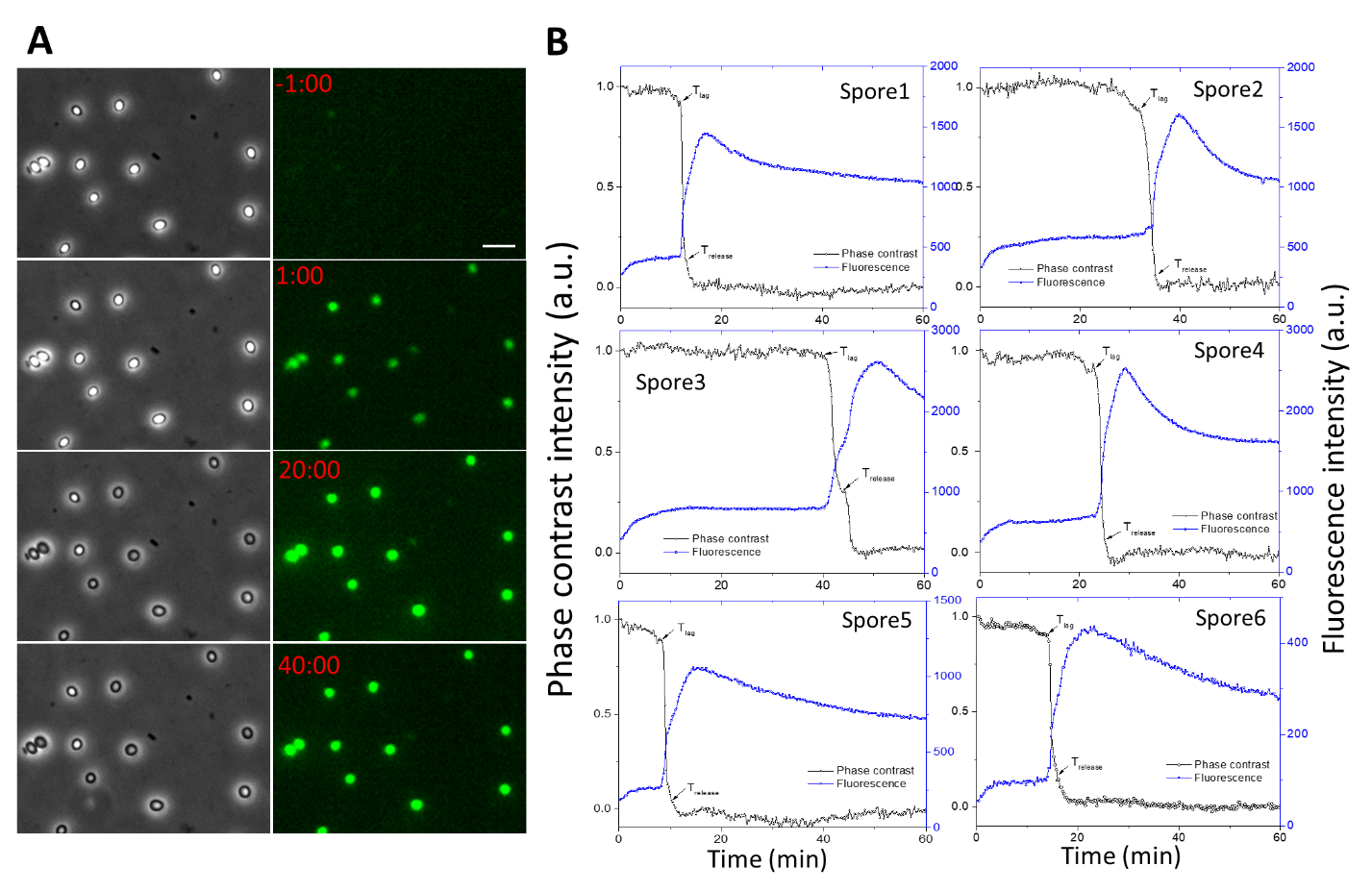
Fig. S7. Germination of multiple individual heat-activated WT *B. megaterium* spores with D-glucose**. **(A)** Phase contrast and fluorescence images at different times before and after addition of D-glucose/ThT as described in Methods. **(B)** Normalized intensities of phase contrast (black curve) and fluorescence (blue curve) images versus incubation time for multiple individual spores - the scale bar is 5 μm. Heat activated spores were germinated with 0.5 mM D-glucose and 10 μM ThT added at time 0 as decribed in Methods. The phase contrast and ThT fluorescence intensities in arbitrary units (a.u.) were normalized to the first image intensity. The arrows in b indicate T_lag_ and T_release_ times, respectively. The arrows in c indicate the T_lag_ times.

Fig. S8.

**
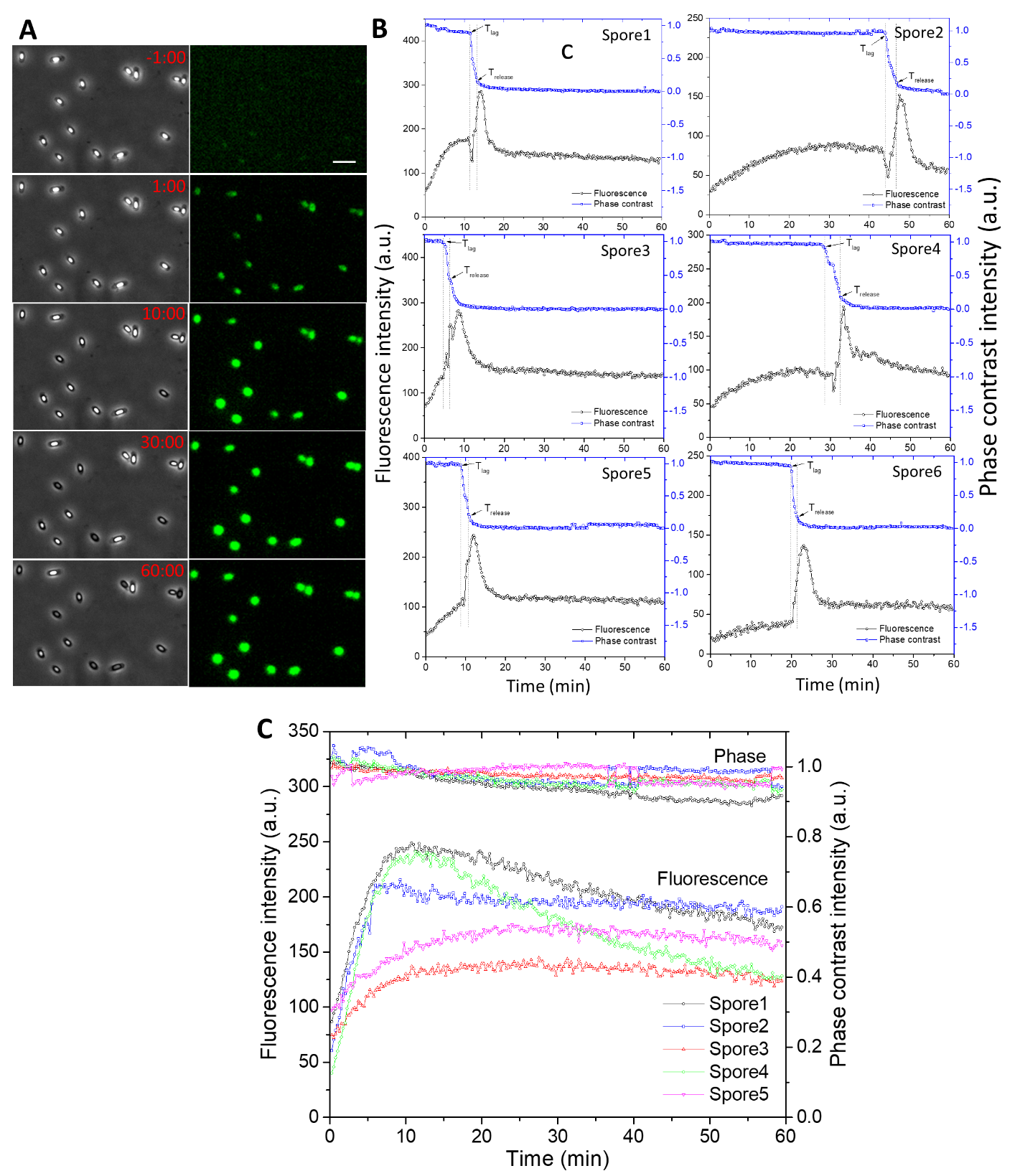
**

**Fig. S8**. **Germination of multiple individual heat activated WT *B. cereus* spores with L-alanine. (A)** Phase contrast and fluorescence images before and after addition of L-alanine/ThT at time 0 were obtained as described in Methods; the scale bar is 5 μm; **(B)** Normalized intensities of phase contrast (blue) and fluorescence (black) images of mutiple individual germinating spores versus time. **(C)** Kinetic changes in phase contrast and ThT fluorescence intensities of multiple individual spores that did not germinate when incubated for 60 min with alanine plus ThT as described in Methods. Arrows and vertical lines in (B) indicate T_lag_ and T_release_ times.

Fig. S9.


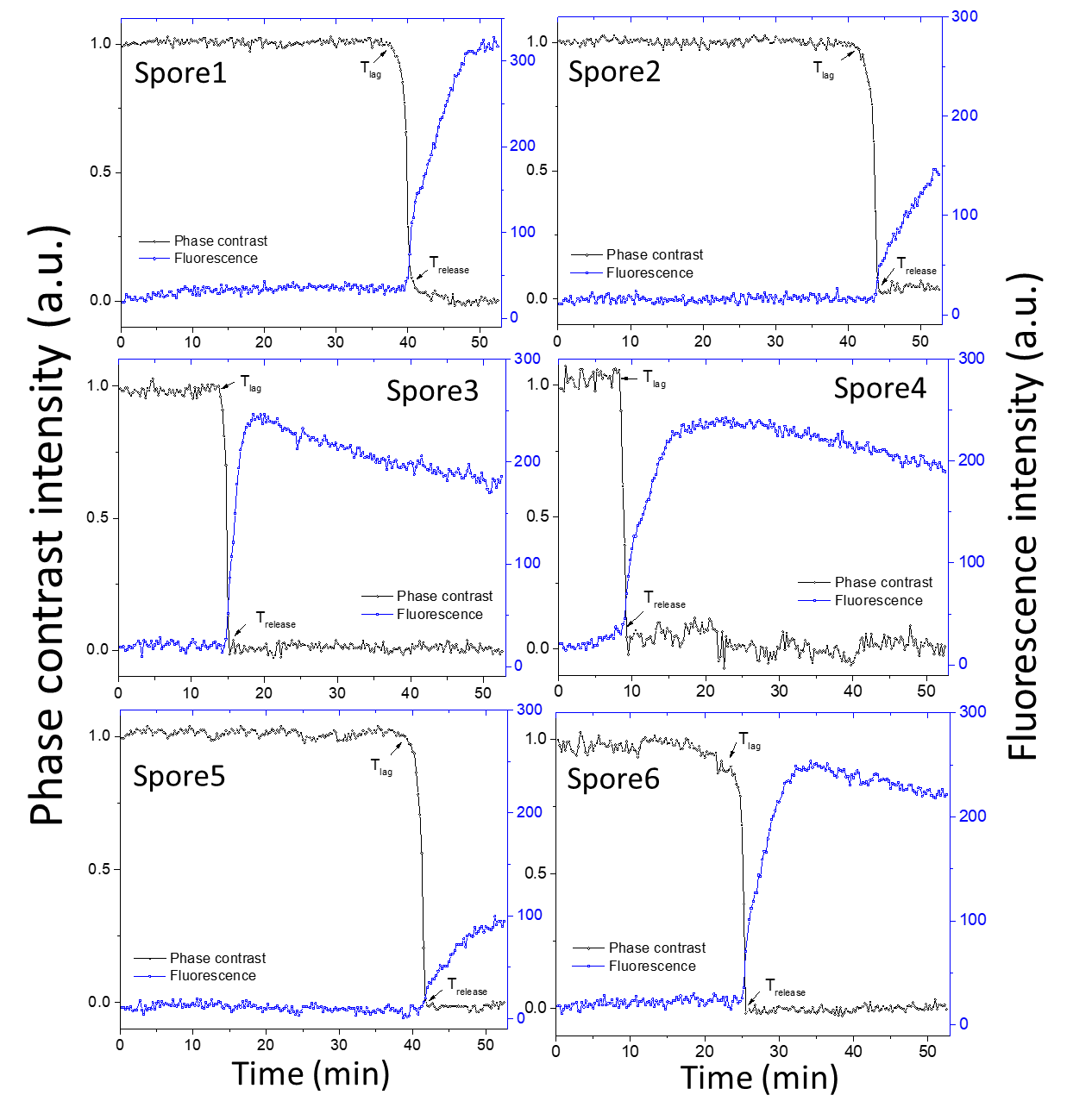
**Fig. S9. Germination of multiple individual WT *B. cereus* spores with dodecylamine**. Spores were germinated in dodecylamine/ThT added at time 0 and observed with phase contrast and fluorescence microscopy as described in Methods. Phase contrast intensities in arbitrary units (a.u.) were normalized to the first image intensity. The arrows indicate T_lag_ and T_release_ times.

Fig. S10.

**
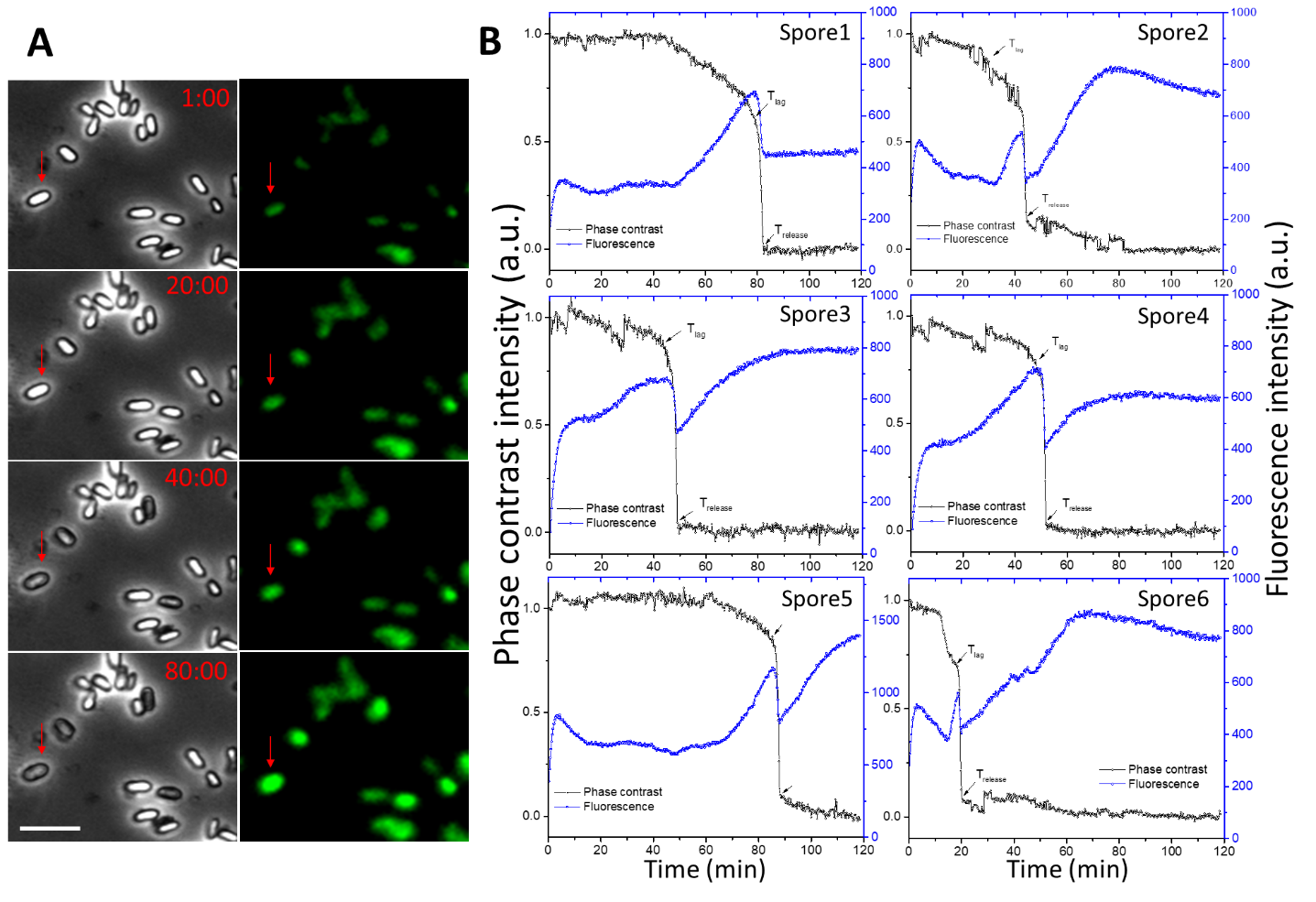
Fig. S10**. **Germination of multiple individual WT *C. difficile* spores with taurocholate/glycine/ThT**. **(A)** Phase contrast and fluorescence images after addition of taurocholate/glycine/ThT at time 0. The red arrows indicate one spore that germinated. **(B)** Normalized intensities of phase contrast and fluorescence images versus time of multiple individual spores. Spores were germinated and observed with phase contrast and fluorescence microscopy, all as described in Methods; the scale bar is 5 μm. The phase contrast intensities in arbitrary units (a.u.) were normalized to the first image intensity. Arrows indicate T_lag_ and T_release_ times, respectively.

**Fig. S11.**

**Fig. S11. ThT accumulation of an individual WT PS832 *B. subtilis* spore** **in ThT-Hepes buffer and during germination with ThT/L-valine**. Changes in normalized ThT fluorescence intensity and phase contrast intensity with incubation time after ThT/Hepes buffer was added prior to time 0. Heated-activated spores were first incubated in distilled water at 37°C and the ThT fluorescence was measured, giving an intensity close to the zero level. At time 0 ThT-Hepes buffer (without germinant) was added and incubated for ~ 30 min, then the ThT-Hepes buffer was removed and replaced by the Hepes buffer for another ~30 min, and then the Hepes buffer was removed and replaced by ThT/L-valine for another ~60 min. The temperature was kept at 37°C and ThT fluorescence images and phase contrast images of individual spores were acquired with a rate of 15s per frame as described in Methods. The T_lag_, T_release_ and T_lys_ timepoints are the start of rapid CaDPA release, completion of CaDPA release and completion of core swelling, respectively.

Movie S1. Germination of multiple individual WT *B. subtilis* (PS832) spores with L-valine/ThT. Heat-activated spores were germinated with L-valine/ThT added at time 0 and examined by phase contrast and ThT fluorescence microscopy as described in Methods. Movie S1 shows time-lapse phase contrast images (left in the movie) and ThT fluorescence images (right in the movie) that were recorded at a rate of 15s per frame for ~60 min.

Movie S2. Germination of multiple individual Coatless *B. subtilis* (PS4150) spores with L-valine. Heat-activated spores were germinated with L-valine/ThT added at time 0 and examined by phase contrast and ThT fluorescence microscopy as described in Methods. Movie S2 shows Time-lapse phase contrast images (left in the movie) and ThT fluorescence images (right in the movie) that were recorded at a rate of 15s per frame for ~60 min.
